# Adult hippocampal neurogenesis shapes adaptation and improves stress response: a mechanistic and integrative perspective

**DOI:** 10.1038/s41380-021-01136-8

**Published:** 2021-05-14

**Authors:** A. Surget, C. Belzung

**Affiliations:** UMR 1253, iBrain, Université de Tours, Inserm, Tours, France

**Keywords:** Neuroscience, Psychiatric disorders

## Abstract

Adult hippocampal neurogenesis (AHN) represents a remarkable form of neuroplasticity that has increasingly been linked to the stress response in recent years. However, the hippocampus does not itself support the expression of the different dimensions of the stress response. Moreover, the main hippocampal functions are essentially preserved under AHN depletion and adult-born immature neurons (abGNs) have no extrahippocampal projections, which questions the mechanisms by which abGNs influence functions supported by brain areas far from the hippocampus. Within this framework, we propose that through its computational influences AHN is pivotal in shaping adaption to environmental demands, underlying its role in stress response. The hippocampus with its high input convergence and output divergence represents a computational hub, ideally positioned in the brain (1) to detect cues and contexts linked to past, current and predicted stressful experiences, and (2) to supervise the expression of the stress response at the cognitive, affective, behavioral, and physiological levels. AHN appears to bias hippocampal computations toward enhanced conjunctive encoding and pattern separation, promoting contextual discrimination and cognitive flexibility, reducing proactive interference and generalization of stressful experiences to safe contexts. These effects result in gating downstream brain areas with more accurate and contextualized information, enabling the different dimensions of the stress response to be more appropriately set with specific contexts. Here, we first provide an integrative perspective of the functional involvement of AHN in the hippocampus and a phenomenological overview of the stress response. We then examine the mechanistic underpinning of the role of AHN in the stress response and describe its potential implications in the different dimensions accompanying this response.

## Introduction

### Adult neurogenesis: a remarkable form of neuroplasticity

For decades, the scientific community acknowledged an immutability of the neuronal architecture of the adult brain. This view can be traced back to the early twentieth century, when the Nobel laureate Santiago Ramon y Cajal [1] summarized his viewpoint as follows: “In adult centers, the nerve paths are something fixed and immutable: everything may die, nothing may be regenerated”. Decades later pioneering studies started to demonstrate empirically the existence of neurogenic niches from which new neurons are generated throughout adulthood in mammals. The dogma started to crack in the 1960s with the identification of adult-generated brain cells in rodents [2]. Following years of skepticism, the neuronal phenotype of new brain cells was eventually confirmed three decades later [3–5].

Adult neurogenesis (AN) has been found in numerous mammals including human and non-human primates [5, 6]. However, it has mainly been limited to two small subareas: (1) the subventricular zone (SVZ) producing new neurons that migrate primarily to the olfactive bulb, and (2) the subgranular zone (SGZ) providing new granule neurons (GNs), the principal neurons in the dentate gyrus (DG) of the hippocampus. The latter is the main, and perhaps the unique source of AN in the human brain [7, 8]. Adult hippocampal neurogenesis (AHN) in humans was revealed in the late 1990s and, since then, confirmed by immunohistochemical identifications [9–12]. However, its occurrence has been disputed by some conflicting results [13, 14], possibly caused by differences in postmortem delay and quality, as well as in tissue processing and histological procedures, as shown by a recent article revealing how slight methodological variations alter the capacity to detect adult-born neurons in the human brain [15, 16]. In addition, AN has also been reported more marginally in a few other brain areas including the striatum, midbrain, and neocortex in different mammal species [17–19]. However, its existence, conditions of occurrence, impact, and source (i.e., SVZ or local) are still debated for these areas [16, 20, 21].

These findings raise the question as to why AN mainly takes place in only a very few restricted subareas of the mammal brain. AN appears to be more widespread in other vertebrates, suggesting that evolution has associated greater complexity of brain anatomy and cognitive functions with structural stability and less neurogenesis [22]. Hence, the question is whether AN in mammals is a functionally relevant singularity or a vestigial remnant of evolution. A hypothetical answer may emerge from issues faced in computational sciences: neural networks require plasticity to process and encode new information, but also stability to preserve unaltered the knowledge previously acquired. This is the so-called plasticity-stability tradeoff dilemma [23, 24]. Transposed to mammal brains, it is easy to understand that ongoing additions of new neurons within the entire brain would result in perpetual network rewiring, compromising previously acquired memories, and supporting a role for AN in forgetting [25]. Accordingly, reducing neurogenesis may have been a means to protect brain functions and memories from catastrophic interference and forgetting, while conserving neurogenesis in restricted subareas may maintain a hint of plasticity to improve the processing and encoding of novel information without altering previously acquired knowledge and memories [26, 27].

### Adult neurogenesis in hippocampal functions

In the hippocampus, neural progenitors located in the SGZ of the DG can proliferate, differentiate into immature adult-born GNs (abGNs) and integrate functionally the granule cell layer and the existing circuitry. All these processes occur under the influence of a large variety of factors acting at different biological levels: network activity, hormones, neurotransmitters, neuropeptides, neuroinflammatory agents, neurotrophic factors, transcription factors, non-coding RNA, and epigenetic modulations [28–31].

The hippocampus has historically been linked to information encoding and processing in relation to two distinctive lines of investigation: (1) spatial mapping, representation of self-location, and navigation on the one hand, mainly through the examination of the properties of place cell activity [reviewed in 32] and (2) episodic memory on the other hand, mainly through the examination of context-dependent learning tasks [reviewed in 33, 34]. More specifically, the hippocampus is now being considered to be pivotal in incorporating unimodal sensory information, temporal sequences, spatial layouts, emotional valences, and combining each of these elements (i.e., “conjunctive encoding”) into coherent multimodal and contextual representations of space and experiences [reviewed in 35–39]. These properties (1) provide the brain with real-time self-localization in allocentric maps [32, 40]; (2) form relational memories of contexts, objects and events [reviewed in 33, 41–43]; (3) index all these elements together in order to retrieve later complete representations from partial cues [44–47]; and (4) guide behaviors by comparing current experience with stored representations and predictions [47–54].

A foremost question is to determine whether AHN is critically involved in one, several, or all aspects of these functions. From a theoretical perspective, adding new neurons endowed with unique cellular properties (e.g., higher excitability, plasticity [55, 56]) at their immature and, at a lesser degree, mature stages [57–59] is believed to enable distinctive computational operations that mature GNs generated during development cannot fulfill, thus optimizing hippocampal functions [26, 60–62]. Research has, however, painted a complex picture in which experimental AHN manipulation does not fundamentally disrupt hippocampal functioning, as most of its neurophysiological properties and functions are essentially preserved under AHN manipulations and none of the typical hippocampus-dependent functions appears to be fully or specifically underlain by AHN [62–67]. For instance, (1) to date no experimental data have highlighted a direct role of AHN in supporting activity of place cells and representation of self-location. There are reports (2) where hippocampus-dependent learning and memory, as well as baseline emotional reactivity and stress-coping behaviors are not, or only marginally, affected by AHN depletion [65–78]; and (3) where general features of the basal neural activity, local field potentials, and synaptic functions in the hippocampus are well conserved following AHN depletion (e.g., basal oscillations, input–output relations, paired-pulse ratio, and synaptic release probability) [68, 79, 80].

However, rather than underlying the full range of hippocampal functions [81], AHN may serve more subtle processes under particular conditions. Consistent observations have indeed demonstrated an instrumental role of AHN in the fine tuning of hippocampal functions that are particularly decisive in terms of behavioral adaptation to environmental demands [reviewed in 61, 82–84]. The abGNs appear to be constantly recruited by hippocampus-dependent tasks, during which they can implement or guide neural activities and computations that drive precise aspects of learning and memory [25, 80, 81, 85–89], as well as responses to stressful experiences [65, 83, 90–93]. In addition, AHN can influence specific neurophysiological features by biasing excitation–inhibition balance and oscillation under particular conditions in the DG [94–99], CA3 and CA1 hippocampal subregions [100], by influencing DG plasticity [101] and providing another form of long-term potentiation depending on GluNR2B-containing NMDA receptors in abGNs [55, 80, 102, 103].

### Adult hippocampal neurogenesis promotes adaptation

The literature has consistently established that, while not being strictly necessary for the encoding and retrieval of hippocampus-dependent memories, AHN promotes the acquisition and precision of the contextual discrimination between overlapping experiences and memories, ultimately enabling individual’s responses to be more appropriately matched with the context [84, 85, 88, 104, 105]. Another part of the literature has highlighted a role of AHN in regulating behavioral and physiological responses to stress. Indeed, exposure to situations that could potentially threaten safety or welfare can engage AHN [reviewed in 82–85]. Under such conditions, AHN might contribute by adjusting emotional reactivity, stress-coping strategy, and neuroendocrine response to the degree of stress estimated from the current circumstances [64, 65, 106, 107].

The literature has therefore divided the functional impact of AHN into two separated dimensions: (1) one related to hippocampus-dependent memory functions, with a strong cognitive layout, (2) another one related to stress response and emotional reactivity (e.g., anxiety, anhedonia), involving strong affective and neuroendocrine components. However, a parallel can be drawn between their presumed outcomes as they are both assumed to promote adaptation (1) to contexts based on previous experiences, optimizing goal-directed behaviors and (2) to stressful conditions, optimizing stress-coping strategies. It is thus conceivable that the role of AHN in memory and its role in the stress response are in fact not independent, representing the two sides of the same coin: adaptation. From this perspective, the unique responsibility of AHN would basically be to modulate hippocampal computations to enable the subject to adapt more appropriately depending on the environmental demands (see section: “Mechanistic underpinnings of adult hippocampal neurogenesis in the stress response”). It is noteworthy that most of the outcomes do not emerge directly within the hippocampus, but through the information conveyed by its projections into extrahippocampal effector structures supporting executive functions, action planning, goal-directed behaviors, emotion, and physiological regulation [82, 108–110].

AHN may therefore appear pivotal in shaping stress responses. Indeed, emotional reactivity, stress-coping behaviors, and neuroendocrine responses (e.g., hypothalamo–pituitary–adrenal (HPA) axis) can be affected by AHN depletion under acute and chronic stress [90–92], although not under all stress conditions [65, 69, 70, 78]. For instance, AHN depletion increased susceptibility to subthreshold social defeat stress (SDS) [92], while it did not exacerbate depression-like phenotypes in the SDS, unpredictable chronic mild stress (UCMS), or chronic corticosterone (CORT) models [65, 69, 70, 78]. However, such an apparent discrepancy might well be explained by a ceiling effect, meaning that the altered phenotypes already reached their apex in the UCMS, CORT, and SDS models and their phenotypes could hardly be worsened by AHN depletion, in contrast to subthreshold procedures. Moreover, both immature and mature abGNs are required to reverse the behavioral effects of chronic stress, to provide resilience to SDS and chronic stress, and to restore an operative hippocampal control over the HPA axis (e.g., strengthening HPA negative feedback) [65, 66, 78, 92, 111–113]. However, it is only recently that studies have started to investigate the mechanistic underpinning of how AHN may regulate the different dimensions of the stress response. Hence, in the next sections we aim: (1) to provide an overview of the current knowledge about the mechanistic underpinning of the functional involvement of AHN in the stress response (“The hippocampus in the stress response” and “The computational impact of adult neurogenesis on the hippocampus”); (2) to decipher the potential functional implications for the different dimensions accompanying stress responses (“Functional involvement of adult hippocampal neurogenesis in the different dimensions of the stress response”). For this purpose, in the next section we provide a phenomenological description of the stress response.

## The phenomenology of the stress response

### History of the notion of stress

The endocrinologist Hans Selye in his seminal paper (1936) showed that after exposure to noxious agents such as “cold, surgical injury, production of spinal shock (…), excessive muscular exercise or intoxications”, animals displayed a similar reaction, whatever the nature of the trigger, that he called a “general adaptation syndrome” [114] and divided into three stages: (1) an “alarm reaction”, corresponding to the acute reaction; (2) a “resistance phase”, appearing when the exposure to the noxious stimulus persisted and the subject tryingly adapts to the condition; (3) an “exhaustion phase”, if the condition persists more than 1 month. This description was concise, centered on acute (alarm reaction) as well as chronic challenging situations (resistance and exhaustion stages), and focused only on physical, but not psychosocial agents. This paper was highly influential, as it pioneered some core concepts that are still considered crucial. Among them, the following are of note: (1) various noxious agents can trigger a similar body response (non-specificity); (2) the response can be either beneficial (termed as “eustress”, which is adaptative) or detrimental, potentially leading to pathologies (“distress”); (3) the beneficial or the detrimental components of the response depend mainly on its duration, as only the chronic situation leads to exhaustion.

Hans Selye is often credited as being the first author to use the term “stress” in the medical literature, but this is not the case: the physiologist Walter Cannon had already used it in a paper in 1935 [115, 116]. The concept of stress draws on another notion proposed by Walter Cannon, that of homeostasis [117]: when stress becomes detrimental, it jeopardizes the homeostasis, that is to say the body’s ability to maintain an internal state stable. Cannon also pioneered the idea that threats to homeostasis can be both physical and psychological [118].

In the 1960s, the psychologist John Mason proposed two fundamental factors [119]: controllability and predictability. Indeed, if the stressors are predictable, for example because the challenges on an organism are repeated, or because a cue is signaling that a stressor is about to be delivered, the subject will progressively show a decrease in its response, denoting adaptation. Furthermore, if the subject can exert control on the delivery or the termination of the stressor, its deleterious impact will be lessened [120–122].

### Stressors and stress response

Currently, the notion of homeostasis and the psychological aspects are well integrated in the literature, as stress is defined as “an actual or anticipated disruption of homeostasis or an anticipated threat to well-being” [123].

**Fig. 1:**
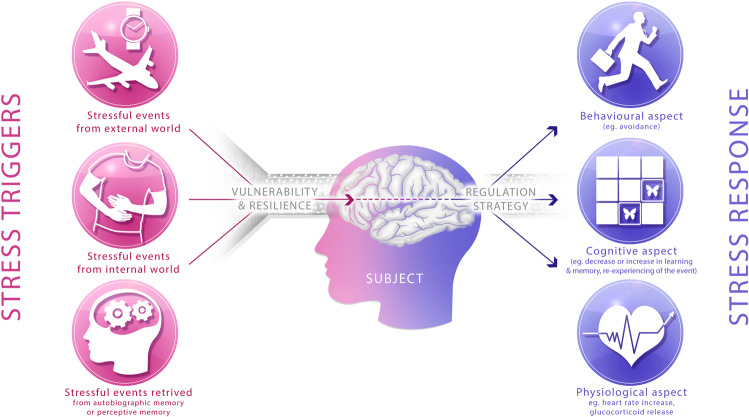
Processing of the stress response. Stressors can either be currently present in the external world or in the internal world (for example visceral pain) but they can also be retrieved from autobiographical memory (past stressful events) or from prospective memory (future stressful events). These stressors will impact on the subjects according to their vulnerability/resilience, and induce a response that includes a behavioral, an affective/cognitive, and a physiological component. These responses can be regulated through different strategies.

Stressful states can be generated from actual events such as stimuli conveyed from the external world through sensory organs or stimuli originating from the interoceptive world [124]. However, it can also be induced by the recall of events stored in the episodic memory [125], or by the anticipation of future stressful events from the prospective memory (Fig. 1) [126]. This last aspect is crucial, as affective disorders such as post-traumatic stress, anxiety disorders, or major depression often originate from stressful stimuli or contexts retrieved from past or anticipated episodic memory [127–129]. Furthermore, stressors can threaten directly the subject’s homeostasis (immediate danger like predator, injury, or inflammation) but also his/her comfort (noisy environment), social status (humiliations, social exclusion, or social isolation), objectives, or introspective thoughts among others.

The response to these stressors includes three components or dimensions: a behavioral, a cognitive/affective, and a physiological dimension (Fig. 1):

1. The behavioral response depends on several factors, such as the proximity to the danger. Indeed, when confronted with a predator the prey can either remain immobile or flee in order to escape or fight if the predator is in close vicinity. The choice of the correct strategy results from assessing the perceived situation. In addition, the subject’s coping strategy can strongly impact the behavioral response. Indeed, a subject can either exhibit an active coping strategy, consisting in seeking further information or requesting help, or display a passive coping strategy consisting in avoiding the situation or remaining immobile [130].
2. The response to the stressful event also induces a subjectively experienced state (negative affect) such as anxiety or distress and also a cognitive component [131, 132]. Concerning the latter, acute stress can enhance or impair cognitive functions. For example, acute stress impacts working memory and cognitive flexibility negatively [133], enhances response inhibition [133], affects decision making [134], enhances processing of high arousing stimuli [135, 136], creates affective bias, stimulates attention, and elicits rumination [137]. Acute stress also has a complex impact on long-term memory that can either be deteriorated or facilitated, depending on the timing and intensity of the stressors [138–140]. Similar effects have been documented regarding extinction, for which the effects of acute stress depend on the timing: if applied before extinction, stress renders memory extinction stronger and less resistant to relapse, while when applied before extinction retrieval, stress impairs extinction and promotes relapse [141].
3. Finally, acute stress also induces a physiological response, consisting in the activation of the sympathetic nervous system (SNS) and the HPA axis culminating in the release of glucocorticoids (i.e., cortisol or corticosterone) as well as in immunological, metabolic, and homeostatic adjustments [142]. In 1988, Sterling and Eyer introduced the term “allostasis” to describe these adaptative physiological response to stressful events [143].

If the stressor is extreme or repeated, subjects can fail to show an adaptive response, resulting in metabolic and psychiatric pathologies such as post-traumatic stress disorder, anxiety disorders, or major depression. In this case, the allostatic response is no longer regulated, compromising the survival of the subject: this situation has been encapsulated in the concept of allostatic load by Bruce Mc Ewen [144]. However, some individuals are able to maintain normal physical and mental functioning in spite of high allostatic load, a phenomenon referred to as resilience. Resilience is a dynamic process that enables the subject to overcome the deleterious consequences of stress [145]. There is, however, a tremendous variability in the population, ranging from highly resilient to highly vulnerable subjects, described extensively and theorized under concepts such as the stress-diathesis model [146]. Finally, the response of subjects to a stressful event also depends on their ability to exert a top-down control over the generation and expression of their emotions, a phenomenon termed as emotional regulation, which enables positive emotions to be increased and negative emotions decreased dynamically via different strategies [147]. Here again, the efficacy of emotional regulation varies greatly among the population [148].

### The neural systems involved in the generation of the stress response

#### Triggers

**Fig. 2:**
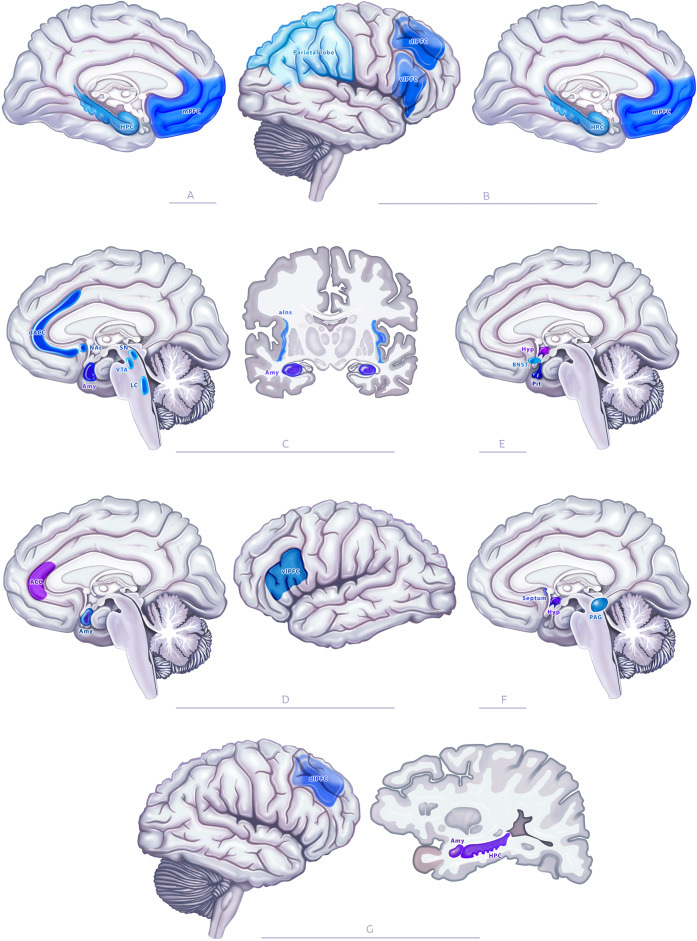
Neural circuits involved in the stress response. The stress response involves networks processing autobiographical (**A**) and prospective memory (**B**), the salience network (**C**), and the regions processing negative valence (**D**), the regions coordinating the physiological response, including the hormonal stress axis (**E**), areas coordinating the behavioral response (**F**), and those processing cognitive/affective (**G**) aspects. HPC hippocampus, mPFC medial prefrontal cortex, vlPFC ventrolateral prefrontal cortex, dlPFC dorsolateral prefrontal cortex, dACC dorsal anterior cingulate cortex, ACC anterior cingulate cortex, NAc nucleus accumbens, Amy amygdala, SN subtancia nigra, LC locus coeruleus, VTA ventral tegmental area, aIns anterior insula, BNST bed nucleus of stria terminalis, Pit pituitary, Hyp hypothalamus, PAG periaqueductal gray.

The stress response is generated and coordinated by several brain networks starting from those processing sensorial information currently arriving from the external world (the thalamus, sensory cortex, and multimodal areas) and those processing information originating from the interoceptive world (the insula and anterior cingulate cortex) [149]. Stressful information can also be retrieved from autobiographic memory, which relies on the medial prefrontal cortex (PFC) and the hippocampus (Fig. 2A) [49–51, 150]. Finally, stress can be the consequence of representations generated from prospective memory, which involves the hippocampus, the parietal, and the PFC (Fig. 2B) [47, 151–154].

#### Evaluation

Stressful information is processed according to two dimensions: its aptitude to elicit arousal and its valence. In general, stressful events elicit high arousal processed by the salience network, and negative valence analyzed by a set of brain areas including the anterior insula, the dorsal anterior cingulate cortex, the amygdala, the nucleus accumbens, the substancia nigra, the locus coeruleus, and the ventral tegmental area (Fig. 2C) [155]. Negative valence is processed by the anterior cingulate cortex, the amygdala, and the ventrolateral PFC (Fig. 2D) [148, 156–159].

#### Response

When valence is negative and arousal is high, the brain will coordinate the stress response, which includes: (1) a physiological response consisting in the activation of the SNS and the HPA axis (Fig. 2E) initiated by the bed nucleus of the stria terminalis (BNST) and the paraventricular nucleus (PVN) of the hypothalamus, respectively [160], stimulating the pituitary gland and inducing the release of glucocorticoids by the adrenals; (2) a behavioral response initiated by subregions of the hypothalamus, the septum, and the periqueductal gray (Fig. 2F) [108, 110, 161]; (3) a cognitive and affective response involving brain areas, such as the amygdala, the hippocampus, and the PFC (Fig. 2G).

### When stress becomes detrimental: the case of chronic stress

**Fig. 3:**
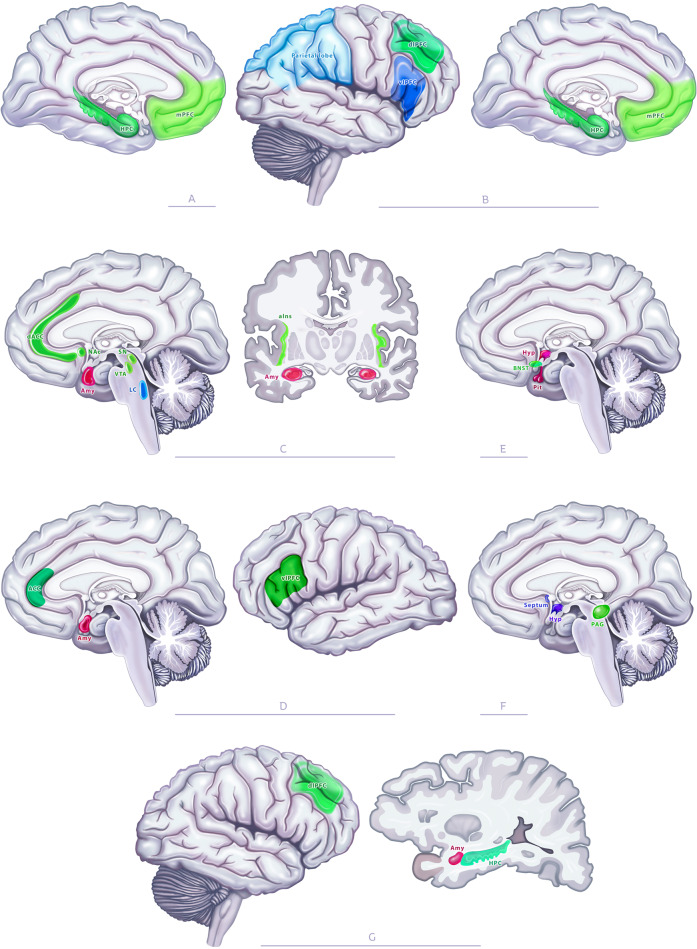
Impact of chronic stress on the neural circuit processing the stress response. Chronic stress impacts the neural circuits processing autobiographical (**A**) and prospective memory (**B**), the salience network (**C**) and the region processing negative valence (**D**), the regions coordinating the physiological response, including the hormonal stress axis (**E**), areas coordinating the behavioral response (**F**), and those processing cognitive/affective (**G**) aspects. Functions whose activity/morphology are unchanged after chronic stress are represented using the same colors as in Fig. 2, regions whose activity is decreased or increased are represented in green and red, respectively. HPC hippocampus, mPFC medial prefrontal cortex, vlPFC ventrolateral prefrontal cortex, dlPFC dorsolateral prefrontal cortex, dACC dorsal anterior cingulate cortex, ACC anterior cingulate cortex, NAc nucleus accumbens, Amy amygdala, SN subtancia nigra, LC locus coeruleus, VTA ventral tegmental area, aIns anterior insula, BNST bed nucleus of stria terminalis, Pit pituitary, Hyp hypothalamus, PAG periaqueductal gray.

The activation of the above-mentioned networks is transient and stops as soon as the stressor has disappeared. However, if the stressor is not removed or is repeated over time, the brain areas involved in the stress response maintain their altered activity, and the SNS and glucocorticoid levels do not return to their basal levels, which can be detrimental. In this case, a functional and structural reorganization of the brain has been observed, leading to a scenario in which some areas become dysfunctional, some exhibiting protracted increased activity, while others decreased activity [162]. Structural changes or modifications in functional connectivity have also been observed. For example, in rodents, chronic stress induces a structural atrophy of the medial and the dorsolateral PFC (Fig. 3A, B, G), the anterior cingulate cortex (Fig. 3C, D), the insula (Fig. 3C), the hippocampus (Fig. 3A, B), the nucleus accumbens (Fig. 3C), the substancia nigra (Fig. 3C), the ventral tegmental area (Fig. 3C), the periaqueductal gray (Fig. 3F), and the BNST (Fig. 3E) associated with increased connectivity between these regions [163, 164]. Other brain areas display hyperactivity and/or hypertrophy. For example, chronic stress induces dendritic hypertrophy in the basolateral amygdala (Fig. 3C, D, g) [165, 166], while the PVN shows hyperexcitability (Fig. 3E) [167]. Finally, chronic stress also leads to changes in outputs from these regions, such as the pituitary (Fig. 3E) that displays increased sensitivity [168] or the hippocampus that displays decreased AHN [65]. Altogether, these changes could alter the functioning of the networks processing the response to acute stress, as the dysfunctional regions are part of the circuits processing, respectively, autobiographic, and prospective memory (Fig. 3A, B, respectively), saliency (Fig. 3C), negative valence (Fig. 3D), coordination of the hormonal stress axis (Fig. 3E), the behavioral response (Fig. 3F), and the cognitive/affective process (Fig. 3G). These dysfunctions recapitulate some of the changes in the brain seen in affective disorders such as major depression or post-traumatic stress disorder [169, 170].

## Mechanistic underpinnings of adult hippocampal neurogenesis in the stress response

### The hippocampus in the stress response

Among the structures described above, figures the hippocampus: it is critically involved in cognitive dimensions of stress, notably through the processing of stress-related information [171, 172]. In addition to the intrinsic properties of the hippocampal circuit (see below), an explanation can emanate from the situation of the hippocampus within the whole brain connectome [173–176]: it receives inputs converging from sensory and association areas allowing the information to be funneled toward the hippocampus. Moreover, hippocampal outputs innervate multiple cortical and subcortical effector areas involved in cognitive, affective, behavioral, and neuroendocrine functions. Consequently, the hippocampus represents a computational hub, ideally positioned to detect cues and contexts linked to stressful experiences and to supervise the expression of the stress response.

Indeed, its potential involvement in stress responses has been increasingly documented [reviewed in 123, 177–180]. Lesional studies have shown that the hippocampus may regulate behavioral dimensions of the stress response including anxiety-like behaviors and emotional reactivity to novelty [181–183], corroborated by optogenetic approaches evidencing a control of anxiety-like and defensive behaviors by the hippocampus, including the DG [108, 184–188]. The hippocampal contribution to the stress response also encompasses the regulation of its neuroendocrine dimension. The hippocampus can stimulate or hamper HPA axis activity via glutamatergic outputs that drive activity into stress-integrative subcortical regions such as the lateral septum, the BNST, and hypothalamic nuclei, which all project into the PVN [65, 123, 189]. Hippocampal neurons (including abGNs) express mineralocorticoid and glucocorticoid receptors influencing the way information related to stress conditions is integrated and consolidated [190–192]. Acute stress can affect hippocampal activity and computation, altering the activity of the principal neurons of the DG, CA3, and CA1 subfields [193–195].

### The computational impact of adult neurogenesis on the hippocampus

The hippocampus can be outlined as a closed computational engine placed at the apex of the brain’s sensory processing stream, with one main gateway (i.e., perforant path), one main exit (i.e., CA1 outputs) between which are hidden layers (DG, CA3, CA1) operating the hippocampal algorithm [33, 173, 174]. In this network, incoming information flows unidirectionally from the entorhinal cortex (EC) via the perforant path and passes through the three main hippocampal subfields DG, CA3, and CA1.

Based on such a connectivity and on its network properties, the hippocampus applies the same general algorithms to all inputs irrespective of their source, nature, or valence, and generates its outputs the same way regardless of the input origin [35]. According to this view, the true intrinsic function of the hippocampus would be merely to execute its algorithm irrespective of any other considerations, and its impact on brain functions would depend only on (1) the origins of the inputs that are processed and indexed together and (2) the effects of the generated outputs on its projection areas. From this perspective, most of the functions classically ascribed to the hippocampus would not be strictly hosted by the hippocampus itself, but might stem from the effects of its outputs.

Accordingly, decoding the precise role of AHN in hippocampal functions, and particularly on stress response, requires understanding its computational role and its influence on the hippocampal algorithm. Within this framework, all the functional impact of AHN must be understood through the prism of its effects on hippocampal computations.

It is noteworthy that the hippocampal connectivity does not supply GNs with any direct extrahippocampal projections: the unique GN outputs are the pyramidal neurons and interneurons of the next hippocampal subfield CA3. This has tremendous implications for the functional impact of AHN, because all the effects of GNs (a fortiori abGNs) on hippocampal computation, behaviors, memory, and stress response must necessarily be mediated by CA3.

The DG and the CA3 perform specialized computations [63, 196–198]. The DG is characterized by expansion recoding and sparse activity [reviewed in 61, 63]. Expansion recoding results in the dilution of the EC inputs into the DG, a wider neuronal layer, each individual GN receiving then only a small fraction of EC information. The sparse activity of GNs originates from their high activation threshold, low firing rate, and strong local inhibitory tone. These properties provide the grounds for “conjunctive encoding” at the cell level and “pattern separation” at the population level [reviewed in 62, 199]. At the cell level, because each GN receives a unique set of EC inputs and requires the summation of several active EC inputs to fire, each active GN conjoins a unique set of contextual elements into a single encoding unit. At the population level, pattern separation corresponds to a computational operation converting parallel input patterns from similar experiences into distinct, orthogonalized output patterns. A representation or a memory trace is assumed to be encoded by a set of firing rates from an active cell ensemble, therefore pattern separation is a process that potentially minimizes interference and disambiguates similar experiences into non-overlapping representations [200, 201].

The CA3 has been postulated to implement “pattern completion” [reviewed in 63, 196, 197, 202]. This property consists in the ability to retrieve a complete, previously stored representation from incomplete, degraded or noisy inputs, thus leading to functions such as memory retrieval, error correction, and generalization. This is made possible by the distinctive neural architecture of CA3, characterized by recurrent collaterals between CA3 pyramidal neurons. This feature endows CA3 with auto-associative (attractor) network properties [33, 197]: incomplete, degraded, or noisy inputs attract CA3 activity into a more stable attractor state embodied by the set of active cells that originally encoded the experience, leading to memory retrieval. Interestingly, attractor dynamics provides the grounds for pattern separation too. When differences between EC input patterns reach a threshold or are provoked by uncorrelated GN discharges, the attractor dynamics cause CA3 activity to fall into a novel attractor state leading to a novel representation.

The abGNs go through a maturation process during which they transiently exhibit a unique connectivity pattern and physiological properties that shape the distinctive way they integrate inputs, respond to incoming information, and influence the hippocampal network [55, 56, 203]. These properties provide abGNs with a “critical period” for 4–8 weeks in rodents during which they can markedly influence local network dynamics and impact DG/CA3 computations as detailed below [61, 204]. In addition, recent evidence indicates that some of the distinctive properties of abGNs may even persist beyond their critical period in certain conditions [58, 205].

**Fig. 4:**
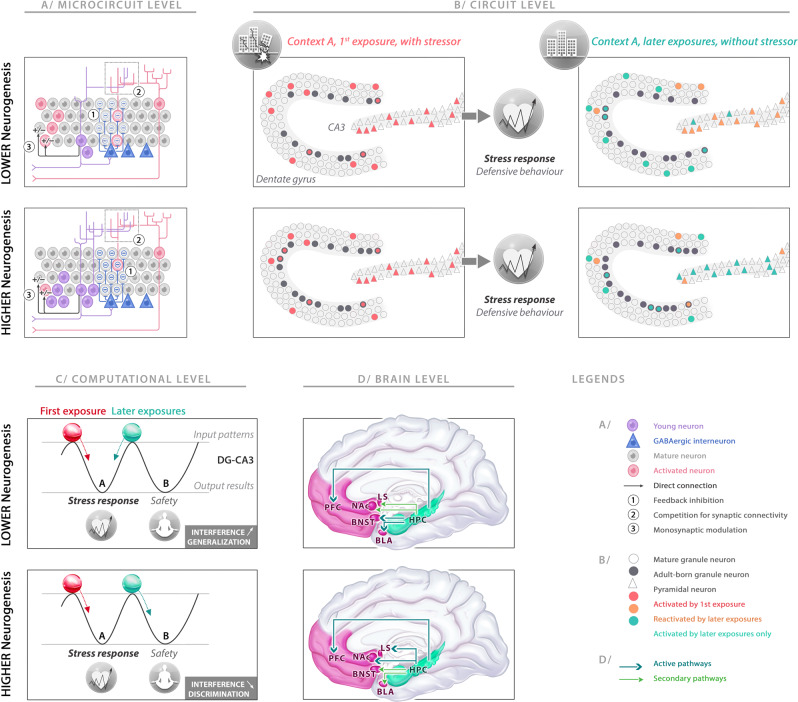
Impact of adult hippocampal neurogenesis (AHN) on response to stress-related context exposure at microcircuit, circuit, computational, and brain levels. **A** Adult-born immature granule neurons (abGNs) in the dentate gyrus (DG) have three major modulatory effects on mature granule neurons (mGNs) at the microcircuit level. (1) Disynaptic feedback inhibition: abGNs can trigger disynaptic feedback inhibition, mediated by local interneurons. By offering a higher feedback inhibitory drive, higher AHN levels promote sparser mGN activity. (2) Synaptic competition: by integrating DG circuitry, the abGN dendrites enter in competition with mGN dendrites for inputs. Higher AHN levels strengthen the destabilization of preexisting synapses on mGNs to the advantage of abGNs, indirectly encouraging pattern separation between older and novel representations. (3) Monosynaptic regulation: the abGNs can bidirectionally gate the inputs to mGNs, positively or negatively depending on whether information comes from the lateral or the medial entorhinal cortex, respectively. **B** At the circuit level, the abGNs can influence DG and CA3 cell ensemble codes during the first exposure to a stressor and the later exposures to a similar context without a stressor. Higher AHN levels induce sparser GN activity but have no impact on the encoding in CA3 ensemble and on the stress response during exposure to a stressor. During a later exposure to a similar context but without a stressor, lower AHN levels result in a relatively new DG ensemble, but not sufficient to impose a new code on CA3, which completes the previous cell ensemble. By contrast, higher AHN levels results in a novel, sparser DG ensemble that contributes to orthogonalizing the active cell ensemble in CA3. **C** The outcomes of the computational operations are represented by two attractor states in the DG-CA3 network one leading to the stress response, the other leading to a new representation associated with safety. The first exposure was associated with a representation (red ball) that felt into the basin of attraction associated with stress response (left). For lower AHN levels, the similarities of the sensory inputs during the later exposures induce the representation (green ball) to fall into the same basin of attraction (left), generalizing the stress response to the second context. With higher AHN levels, modulatory effects of abGNs on DG and CA3 help to uncorrelate the incoming information from the original experience and provoke the representation to fall into the other basin of attraction (right), reducing interference and leading to a new representation associated with safety (context discrimination). **D** The hippocampus processes the information, performs its computational operations under the influence of AHN levels and then supervises downstream effector regions including the prefrontal cortex (PFC: memory, affective evaluation and cognitive flexibility), lateral septum (LS: regulating defensive behaviors), nucleus accumbens (NAc: valence and salience processing), bed nucleus of stria terminalis (BNST: coordinating neuroendocrine response), and basolateral amygdala (BLA: affective and valence processing). The hippocampus sends its instructions to these structures for a coordinated stress response, promoting or dampening the functions of each area depending on the outcome of its computational operations.

1. AHN enables new units with distinct encoding capacities to be inserted into the DG. During their critical period, the abGNs are more prone to fire in response to EC inputs and therefore it is unlikely that abGNs directly subserve DG pattern separation [204, 206]. These physiological characteristics are profitable for conjunctive encoding, leading authors to posit that abGNs act as pattern integrators [27]. Hence, by tending to link disparate elements of a context, abGNs would function as conjunctive cells [207]. The abGNs are indeed more active and less spatially-tuned than mature GNs (mGNs) during active explorations [208], consistent with conjunctive encoding.
2. Another putative role supported by AHN concerns modulatory effects on both mGNs in the DG and pyramidal neurons in the CA3. Indeed, in addition to innervating CA3 pyramidal neurons [209], abGNs form synapses with DG/CA3 interneurons enabling abGNs to trigger disynaptic feedback inhibition on mGNs [94, 210], and disynaptic feedforward inhibition on CA3 pyramidal neurons [210, 211]. Higher AHN levels are indeed linked to higher inhibitory drive on mGNs [94, 97]. By consequence, the activity of mGNs in the DG is sparser (Fig. 4A). Higher AHN levels would also modify the excitability of CA3 pyramidal neurons, leading to higher feedforward inhibitory tone maintaining sparse CA3 activity. Hence, despite their enhanced excitability, the abGNs exert modulatory effects that promote sparse coding and pattern separation in the DG-CA3 network.

(4) Another indirect modulatory effect originates from synaptic competitions for the DG afferences (including EC inputs) between abGNs and mGNs (Fig. 4A). Indeed, to integrate local circuits, the abGNs have to extend their dendritic arborescence into the molecular layer that is accompanied by rewiring and elimination of preexisting synapses of mGNs and older abGNs [212, 213], potentially destabilizing previous representations, favoring the formation of new neuronal ensembles, and thus indirectly promoting pattern separation.
(5) A last modulatory effect stems from the transient functional synapses that abGNs form directly on mGN dendrites. abGNs can bidirectionally gate the inputs to mGNs (Fig. 4A) depending on the source of the information they receive [214]. Indeed, in response to lateral EC inputs (relaying item- and context-related information), abGNs exert a monosynaptic inhibition on mGNs via metabotropic glutamate receptors while in response to medial EC inputs (which relay spatial information) abGNs monosynaptically stimulate mGNs via GluN2B-containing NMDA receptors. Accordingly, the abGNs have wide latitude to fine-tune mGN activity via both monosynaptic modulation and disynaptic feedback inhibition. Both types of regulation may coexist in the DG, but they follow different temporal dynamics with the direct monosynaptic modulation of mGNs being established immediately prior to or at the beginning of the critical period (abGNs aged 4 weeks) [214], and the onset of disynaptic feedback inhibition appearing during the critical period of abGNs and their transition toward maturity (abGNs aged ≤7weeks) [94, 210].

Altogether, these properties provide abGNs with great flexibility and the ability to finely retune DG/CA3 neural representations.

Thus abGNs transiently act as pattern integrators binding together the elements of an experience. Once mature, these cells would become less active and less inclined to be enrolled in novel representations than novel abGNs (even if keeping higher inclinations to be recruited than older mGNs generated during development, see [58, 205]). It is possible that this process helps linking memories in time [215]. Successive waves of abGNs would facilitate the formation of more separated CA3 representations at different times, even for similar experiences and contexts. Accordingly, immature abGNs being excitable conjunctive cells that integrate very different elements of a context at one time point, would still be able to subserve pattern separation but on different timescales (“*temporal pattern separation*”): current versus previously acquired (or predicted) representations [216].

As indirect modulators, it is assumed that the net effect of abGNs is to facilitate the orthogonalization of CA3 representations for low input differences (Fig. 4B), reducing overlaps between distinct CA3 cell ensemble codes (not necessarily the DG [217]), thus facilitating discrimination between different contexts or spatial layouts [88, 104] and reducing interference between different representations in the same temporal window (“*spatial pattern separation*”) [218 but see 219]. This may result in gating downstream hippocampal regions with more accurate and contextualized information, enabling behavioral responses to be appropriately matched to a specific context, which is particularly adaptive for stress-related conditions (Fig. 4C, D). Indeed, promoting spatial pattern separation is believed to contribute to coping with stressful situations and to prevent generalization of stressful experiences to safe contexts [83, 93].

From this mechanistic basis, it is assumed that the impact of AHN is to bias hippocampal computations toward pattern separation in both narrow-time windows as a modulator (*spatial pattern separation*) and over broader timescales as an encoding unit (*temporal pattern separation*). From a more general perspective, it provides an instrumental role of AHN in the fine tuning of hippocampal functions under high interference conditions promoting: rapid and flexible acquisition of new contextual representations, specificity and precision of hippocampal representations, reduction of proactive interference during conflict resolution and uncertainty, avoidance of overgeneralizing fear and acceleration of system consolidation and indexing [82–84, 220, 221]. All these properties positively influenced by AHN are assumed to promote together adaptation, cognitive flexibility, and optimized response to stressful situations.

### Functional involvement of adult hippocampal neurogenesis in the different dimensions of the stress response

In the previous sections, we described the processing of information that may be related to the stress response, including how it is generated, the response produced to deal with it and how it is modulated by the vulnerability/resilience of the subject. The question remains as to how the impact of AHN on hippocampal computations reverberates through other dimensions of the stress response.

#### Triggers/evaluation

In previous sections, we detailed how the stress response can be generated either from actual events occurring in the surrounding environment of the subject, or from internal triggers, or from past or future representations (Fig. 1). To our knowledge, no study investigated a causal role of abGNs in processing interoceptive states, even if the DG seems involved in it in a more general way [222]. However, the above-described function of abGNs indicates that it will directly participate in the processing of external triggers, as well in the assessment of triggers generated from the past or anticipated episodic memory. Indeed, (1) it will render the information from external triggers more accurate and contextualized, thus facilitating a response adapted to the actual situation, avoiding non adapted over-general responses (see above section). The generation of the stress response will thus be more precise, occurring only in specific situations, thus enabling it to occur less frequently. This might in part explain why enhancement in the number of abGNs is dampening the effects of chronic stress: the addition of abGNs to the network, by decreasing the frequency of the stress response, might thus reduce the number of events inducing a response, and consequently, the impact of chronic stress on the behavior of the subject and on its brain. (2) It will facilitate retrieval of stressful information from memory in order to construct predictions, functions in which the hippocampus, and notably AHN participate. Indeed, AHN contributes to contextual fear memory [82, 85, 223], which indicates that it might alter the capacity to encode and remember aversive events, even if those events were not present in the current context. Furthermore, its involvement in context discrimination and in pattern separation might also alter aversive memories [64, 88, 104]: a lower level of AHN might decrease discrimination between negative and safe events, inducing a generalization of the stress response even to innocuous situations [83, 93, 224]. A direct role of AHN in prospective memory has not been demonstrated yet. However neurophysiological evidence indicates that cells within the CA3 and CA1 regions of the hippocampus are involved in sharp-wave ripple-associated awake replays, a process closely related to the organization of future behaviors [47–50, 225, 226], including memory-guided behaviors for reward seeking and stressful/aversive cue avoidance [51, 227, 228]. Interestingly, this process has recently been found in the DG too, and may implicate abGNs [229, 230]. Although to date, abGN involvement has only been suggested from reactivated ensembles during sleep, these findings support the possibility that AHN contributes to the anticipation and avoidance of future stressful events, an effect that would promote predictability and controllability of the stressors.

#### Response

As depicted above, the stress response consists of three components: a behavioral, an affective/cognitive, and a physiological one. While AHN might not participate per se in generating the behavioral aspects of the stress response, which is rather orchestrated by hypothalamic and hindbrain neural circuits, it seems largely involved in regulating its affective, cognitive, and physiological dimensions.

Modifying the number of abGNs or their activity impacts anxiety-like responses and coping strategies in rodent behavioral bioassays (e.g., tail suspension test, elevated plus-maze, predator stress….) [90, 112, 231], emotional learning [107, 208, 223], cognitive flexibility [84, 232, 233], and working memory [219, 233]. Furthermore, it can also be speculated that AHN is involved in behavioral inhibition, and attention, as the hippocampus contributes to these functions [234, 235], even though direct evidence of the role of AHN remains to be demonstrated [236].

AHN also impacts directly on the physiological aspect of the stress response. Indeed, while a direct function of AHN in regulating the stress-induced activation of the SNS has not been really demonstrated, large evidence shows that it is involved in the regulation of the HPA axis. Indeed, although abGN involvement in the fine tuning of the HPA axis under basal conditions is still controversial, as contradictory results depending on timing (day versus night) and conditions (acute stress or not) have been described [237], the impact of AHN in the case of individuals subjected to unpredictable chronic stress seems clear [65, 91, 238, 239]. Indeed, in the case the two systems that normally collaborate to regulate the HPA axis (the PFC and hippocampus) are damaged, the increase of AHN is necessary to restore a normal regulation of the HPA axis under chronic stress [65].

Such functions in stress response appear to engage primarily the ventral part of the hippocampus and AHN. Indeed, a functional dissociation along the dorso-ventral axis paralleled with distinct connectivity patterns have consistently been emphasized, supporting a more critical role of the ventral hippocampus in processing emotional and stress-related information [92, 240].

#### Vulnerability—resilience

Finally, AHN is also crucial to stress resilience/vulnerability. For example, increasing AHN, specifically in the ventral hippocampus, promotes stress resilience while chemogenetic inhibition of abGNs confers vulnerability. This effect has been suggested to occur via modulation of stress-responsive cells located in the ventral part of the DG and projections of the ventral CA1 to stress-responsive areas of the brain [92, 240].

Taken together, this shows that abGNs might be causally involved in most aspects of the stress response, as increasing their number or their function will enable (1) a more accurate response to stimuli from the actual environment or retrieved from memory, (2) a more relevant affective, cognitive, and endocrine response, (3) an increased resilience. Its increase will thus reduce the detrimental consequences of chronic or repeated stressful experience, as it will both decrease the number of events triggering a stress response, facilitate an adapted affective and cognitive response, promote a better regulation of the HPA axis, which will in turn reduce the detrimental impact of glucocorticoids on brain structures and function. Therefore, the stimulation of abGNs might reduce the psychopathological and pathophysiological consequences of chronic stress [241].

## Conclusion

We have reviewed theoretical and experimental work whose combined findings strongly support a modulatory role of AHN in the stress response. In an integrative approach, we have considered that AHN is pivotal in shaping adaptation to demanding environments. These effects are assumed to rely on AHN influences on the computational operations performed by the hippocampus and to be conveyed by a hippocampal top-down control over downstream brain areas involved in the expression of the stress response at the behavioral, affective, cognitive, and physiological levels.

It is noteworthy that both CA2 and subiculum subareas have not been described in the present review, as knowledge regarding their specific computational roles is still scarce so far and because it is beyond the scope of this review to enter into such network details and speculate on their role. Indeed, CA2 is a subfield located between CA3 and CA1 that has long been ignored by research as it is a diminutive area that displays strong anatomical similarities to CA3, while subiculum represents a supplemental exit subfield right after CA1. However, in the near future it might be of great value to better characterize their specific computational roles in order to estimate how they may distinctly contribute to mediating abGNs effects on downstream structures.

From a clinical perspective, in individuals subjected to chronic or traumatic stress, AHN would be disrupted together with other brain areas involved in evaluating and regulating emotions, potentially resulting in affective neuropathologies. As an indirect modulator of larger brain areas, AHN represents a promising target to trigger recovery. However, as AHN acts through its computational properties that mainly operate regardless of the nature and the valence of the incoming information, future work should test whether the beneficial consequences of manipulating AHN over maladaptive stress responses are not neutralized by the possible detrimental results on positive memories and other adaptive responses.
